# Kangaroo Mother Care in Developing Countries With Poor Hygiene or Limited Healthcare Infrastructure: A Systematic Review

**DOI:** 10.7759/cureus.101779

**Published:** 2026-01-18

**Authors:** Raghu Nandan Reddy B, Sunil Kumar, Syed Shah Naseeruddin Sarmast, Mukeeb Ahmed Mohammed, Rakesh Kotha

**Affiliations:** 1 Pediatrics, Mahavir Institute of Medical Sciences, Vikarabad, IND; 2 Neonatology, Osmania Medical College, Hyderabad, IND

**Keywords:** hygiene practice, kangaroo mother care (kmc), low-resource settings, neonatal infections, neonatal mortality and morbidity, skin-to-skin contact

## Abstract

Kangaroo Mother Care (KMC) is an affordable, evidence-based intervention widely endorsed for improving survival and health outcomes in preterm and low-birth-weight infants, particularly in resource-constrained healthcare settings. Concerns persist regarding its use in areas with suboptimal sanitation, mainly due to fears of increased newborn infection rates. This systematic review evaluates KMC's effectiveness and safety in contexts characterized by poor hygiene and limited resources. Using the PICO framework, we conducted comprehensive searches across PubMed, Cochrane Library, Scopus, Web of Science, and Google Scholar for publications from January 2005 to July 2024. Eligible studies included randomized controlled trials, cohort studies, and systematic reviews reporting mortality or infection outcomes. Quality was assessed using Cochrane Risk of Bias tools and AMSTAR (A Measurement Tool to Assess Systematic Reviews) 2. Due to heterogeneity, a narrative synthesis was performed rather than a meta-analysis. The evidence consistently demonstrates reductions in neonatal mortality, along with improvements in breastfeeding rates and thermoregulation, even in poor sanitary conditions. No overall increase in severe infections was observed; sepsis rates often decreased. Basic preventive measures (e.g., hand hygiene) effectively mitigate any residual risks. These findings confirm that KMC is appropriate and safe for low-hygiene settings when combined with simple precautionary practices.

## Introduction and background

Neonatal mortality rates remain substantially elevated in low- and middle-income countries, primarily due to inadequate medical facilities, overcrowded living conditions, and suboptimal sanitary standards [1,2]. Preterm or low-birth-weight infants are particularly vulnerable to hypothermia, bloodstream infections, and feeding difficulties; these issues contribute to a significant proportion of preventable neonatal deaths [3].

Kangaroo Mother Care (KMC) involves prolonged skin-to-skin contact between the caregiver and infant, promotion of exclusive breastfeeding, and early discharge from facilities with continued support. Research has shown that KMC improves survival rates and supports long-term development in this vulnerable population [4,5]. Leading global health organizations recommend KMC as standard care for physiologically stable preterm newborns, particularly in settings lacking advanced technology [6]. However, concerns persist about its use in low-sanitation areas due to potential risks from contaminated water, unclean materials, and limited caregiver education on infection prevention [7,8].

Despite these concerns, KMC is a practical and cost-effective intervention ideally suited to resource-constrained health systems [9]. This review synthesizes current evidence on KMC's effectiveness and safety in environments with poor hygiene and limited resources, focusing particularly on neonatal survival and infection outcomes.

## Review

Aim

This systematic review aims to assess the effectiveness and safety of KMC in developing countries, particularly in settings with poor sanitation and limited healthcare resources, focusing on neonatal mortality and infection-related outcomes.

Objectives

This review evaluates KMC's impact on neonatal mortality and infection outcomes in developing countries, examines secondary outcomes such as breastfeeding success and thermoregulation, and identifies evidence gaps for implementation in low-hygiene environments.

Methods

PICO Structure

Using a PICO framework, the population comprised full-term and preterm neonates in settings with inadequate sanitation and limited healthcare resources; the intervention was KMC involving continuous skin-to-skin contact between the caregiver and newborn; the comparator was conventional neonatal care in hospital or facility-based settings without structured skin-to-skin contact; and the outcomes included neonatal mortality, infection-related outcomes, breastfeeding success, and thermal stability.

Search Approach

We conducted a comprehensive literature search across PubMed, the Cochrane Library, Scopus, Web of Science, and Google Scholar for studies published between January 2005 and July 2024. The search strategy combined Medical Subject Headings (MeSH) and free-text terms using Boolean operators. Synonymous terms within each concept were combined using OR, including intervention-related terms ("kangaroo mother care" OR "kangaroo care" OR "skin-to-skin contact"), outcome-related terms (neonatal mortality OR infant mortality OR infant death OR infection OR sepsis OR breastfeeding OR thermoregulation OR thermal stability), and setting-related terms ("Developing Countries" (MeSH) OR developing countr* OR low-income countr* OR middle-income countr* OR LMIC* OR resource-limited). These concept groups were then combined using AND to identify studies addressing KMC in relevant settings with reported clinical outcomes. The search was limited to human studies published in English. Reference lists of included studies and relevant reviews were manually screened to identify additional eligible articles.

Inclusion and Exclusion Criteria

Studies were eligible if they were randomized controlled trials, cohort studies, or systematic reviews conducted in low- or middle-income settings and reported neonatal mortality or infection outcomes associated with KMC. Case reports, small case series (fewer than 10 participants), non-English publications, and studies conducted exclusively in advanced neonatal intensive care units in high-income countries were excluded.

Study Selection and PRISMA Flow

Initial searches yielded 1,250 records. After removing 330 duplicates, 920 records underwent title and abstract screening; 820 were excluded as unrelated to KMC, lacking focus on limited resources/sanitation, or not involving neonates.

**Figure 1:**
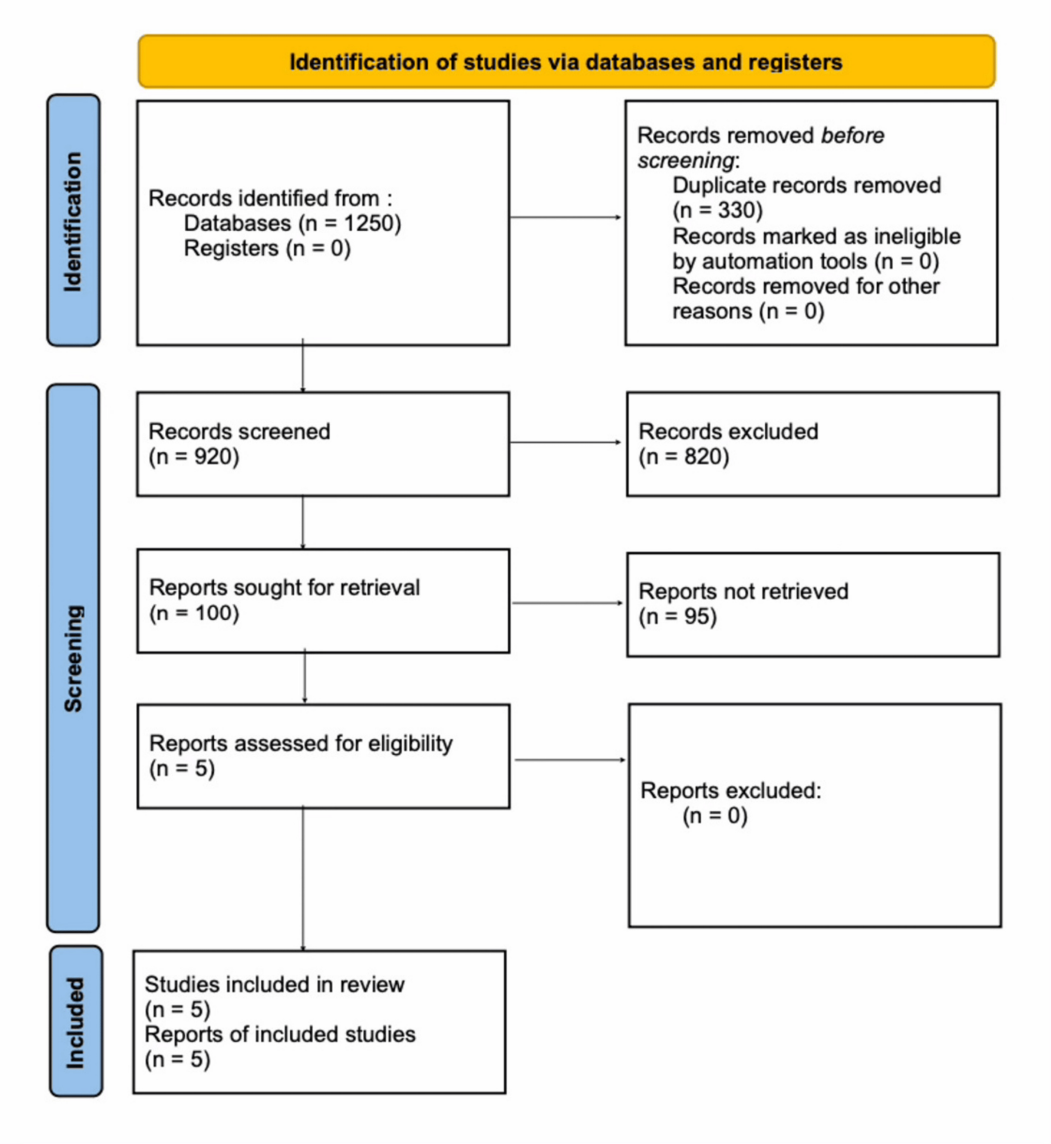
PRISMA flow diagram PRISMA, Preferred Reporting Items for Systematic Reviews and Meta-Analyses

Of the remaining 100 records, full texts were retrieved. Five studies met the inclusion criteria and were included in the qualitative synthesis. All five passed detailed eligibility checks and underwent an in-depth narrative review. Selection followed Preferred Reporting Items for Systematic Reviews and Meta-Analyses (PRISMA) guidelines for systematic reviews (see Figure 1). The protocol was prospectively registered with PROSPERO (CRD420251118736). Included studies were from low- and middle-income countries in Africa and Asia.

Results

Quality Assessment

We assessed risk of bias in the randomized trials using the Cochrane Risk of Bias Tool. Systematic reviews were evaluated with AMSTAR (A Measurement Tool to Assess Systematic Reviews) 2. Overall, the included studies demonstrated moderate to high methodological quality (Table 1).

**Table 1:** Quality assessment of included studies AMSTAR, A Measurement Tool to Assess Systematic Reviews

Study (Citation)	Study Design	Assessment Tool	Key Quality Domains Assessed	Overall Risk of Bias / Quality
Conde-Agudelo and Díaz-Rossello (2016) [4]	Systematic review and meta-analysis	AMSTAR 2	Comprehensive literature search, explicit inclusion criteria, appropriate synthesis methods, assessment of publication bias	High quality
Boundy, et al. (2016) [5]	Meta-analysis	AMSTAR 2	Robust study selection, transparent outcome reporting, sensitivity analyses	High quality
WHO Immediate KMC Study Group (2021) [10]	Multicenter randomized controlled trial	Cochrane Risk of Bias Tool	Random sequence generation, allocation concealment, attrition, outcome reporting	Low risk of bias
Mazumder, et al. (2019) [11]	Cluster randomized controlled trial	Cochrane Risk of Bias Tool (cluster-adapted)	Randomization, incomplete blinding, outcome assessment, attrition	Moderate risk of bias
Sivanandan, et al. (2023) [12]	Systematic review and meta-analysis	AMSTAR 2	Comprehensive search strategy, clear eligibility criteria, risk-of-bias assessment, publication bias	High quality

The five included studies comprised three systematic reviews/meta-analyses and two randomized controlled trials, conducted predominantly in low- and middle-income countries with variable hygiene standards (Table 2). These studies evaluated large cohorts, with sample sizes ranging from over 3,000 to more than 15,000 neonates. The evidence consistently demonstrated significant reductions in neonatal mortality (relative risks ranging from 0.60 to 0.78) and in the risk of severe infection or sepsis (relative risks ranging from 0.50 to 0.85). Importantly, none of the studies reported an increased risk of infection associated with KMC in low-hygiene settings. Several studies further emphasized that adherence to basic hygiene practices played a protective role in sustaining these benefits across both hospital- and community-based settings.

**Table 2:** Summary of included studies LMICs, low- and middle-income countries; KMC, Kangaroo Mother Care

Study	Country / Setting	Study Design	Sample Size	Hygiene Context	Key Findings
Conde-Agudelo and Díaz-Rossello (2016) [4]	Multiple LMICs	Systematic review and meta-analysis	>3,000 low-birth-weight neonates	Variable, predominantly low resource	KMC reduced neonatal mortality (RR 0.60) and severe infection/sepsis (RR 0.50); consistent benefits
Boundy, et al. (2016) [5]	LMIC hospitals and communities	Meta-analysis	15,679 neonates	Poor sanitation common	Reduced mortality (RR 0.64) and sepsis (RR 0.53); no increase in serious infections
WHO Immediate KMC Study Group (2021) [10]	Multiple LMICs (India, Africa)	Multicenter randomized controlled trial	3,211 preterm neonates	Low-resource hospital settings	Reduced mortality (RR 0.75) and sepsis (RR 0.82)
Mazumder, et al. (2019) [11]	Rural India	Cluster randomized controlled trial	8,402 low-birth-weight neonates	Poor community hygiene	Reduced mortality (aRR 0.78); no increase in infection rates
Sivanandan, et al. (2023) [12]	Multiple LMICs	Systematic review and meta-analysis	15,559 neonates	Predominantly low resource	Reduced mortality (RR 0.68) and severe infection (RR 0.85)

Conde-Agudelo and Díaz-Rossello (2016)

This Cochrane systematic review reported a reduction in neonatal mortality with KMC (RR 0.60, 95% CI 0.39-0.92) and a lower risk of severe infection, including sepsis (RR 0.50, 95% CI 0.36-0.69) [4]. These effects were observed across low- and middle-income countries with varying hygiene standards. Infection outcomes were not uniformly defined across included trials, limiting hygiene-specific subgroup analyses; however, the review found no evidence of increased infection-related mortality in low-hygiene settings [4]. Overall, these findings support the safety of KMC even in contexts with limited sanitary infrastructure, with no pattern of increased harm apparent in poorer sanitary conditions.

Boundy, et al. (2016)

This meta-analysis, including 15,679 neonates, found that KMC was associated with a significant reduction in neonatal mortality (RR 0.64, 95% CI 0.46-0.89) [5]. The analysis also demonstrated higher rates of exclusive breastfeeding and a lower incidence of neonatal sepsis among infants receiving KMC (RR 0.53, 95% CI 0.40-0.69). Study settings varied widely in sanitation and resource availability; however, no increase in infection-related mortality was observed. Although inconsistent documentation of hygiene practices prevented detailed subgroup comparisons, the overall results remained favorable. Collectively, these findings suggest that the benefits of KMC persist across diverse care environments, including those with limited sanitary infrastructure [5].

WHO Immediate KMC Study Group (2021)

This multicenter randomized controlled trial, conducted in low-resource hospitals across multiple low- and middle-income countries, reported a reduction in neonatal mortality with immediate KMC (RR 0.75, 95% CI 0.64-0.89) [10]. A trend toward reduced sepsis was also observed (RR 0.82, 95% CI 0.67-1.00) [10]. Reported infections were localized and not associated with increased mortality. The study demonstrated the feasibility of implementing KMC in facility-based settings with limited resources [10]. These outcomes indicate that early initiation of KMC can be effectively integrated into routine neonatal care despite infrastructural constraints, and concerns related to infection risk do not outweigh the observed survival benefits.

Mazumder, et al. (2019)

This cluster randomized controlled trial, conducted in rural India, reported reduced neonatal mortality with community-based KMC (adjusted RR 0.78, 95% CI 0.66-0.92) [11]. No increase in infection rates was reported. The study took place in community settings with limited hygiene infrastructure, and its randomized design strengthens the validity of the mortality findings [11]. These results demonstrate that KMC can be effectively delivered outside hospital environments and suggest that community-level implementation does not inherently elevate infection-related risks.

Sivanandan, et al. (2023)

This systematic review and meta-analysis, including 15,559 neonates, reported reductions in neonatal mortality associated with KMC (RR 0.68, 95% CI 0.53-0.86) and in severe infection (RR 0.85, 95% CI 0.79-0.92) [12]. The authors emphasized the importance of basic hygiene measures when implementing KMC in low-resource settings [12]. Beneficial effects were observed across included studies despite variations in care environments. These findings indicate that simple supportive practices can accompany KMC without diminishing its effectiveness.

Discussion

This systematic review summarizes evidence indicating that KMC is associated with reduced neonatal mortality in low-hygiene and resource-limited settings. Across the included studies, KMC consistently demonstrated improved survival rates as well as benefits in breastfeeding success and thermoregulation in settings with limited healthcare infrastructure [4,5,9].

Concerns about infection risk have been cited as a potential barrier to KMC implementation in areas with poor sanitation. However, none of the included studies reported an overall increase in serious infections associated with KMC, and several documented reductions in sepsis [4,5,10]. Where described, basic hygiene measures (e.g., hand hygiene, use of clean wraps, and caregiver education) were implemented alongside KMC [6,13].

The findings of this review align with those from community- and facility-based KMC studies in India and Africa, which reported reductions in neonatal mortality without an increase in infection-related deaths [11,14]. Broader analyses have also highlighted KMC as a cost-effective intervention in low-resource settings [3,15].

In addition to clinical outcomes, several biological mechanisms may explain why KMC does not increase infection risk in low-hygiene settings. Prolonged skin-to-skin contact promotes close maternal-infant interaction during a critical window of immune development. Exposure to maternal skin microbiota supports early microbial colonization essential for immune maturation, particularly in rural and resource-limited environments, as shown in population-based microbiome studies [16-18]. Although microbiome-specific outcomes were not directly assessed in the included studies, existing evidence on early microbial development provides biological plausibility for the observed lack of increased severe infections across diverse low-resource settings [18,19].

Breastfeeding promotion is another key component of KMC that influences infection-related outcomes. Skin-to-skin contact facilitates early initiation and sustained exclusive breastfeeding, which delivers immunological protection through antibodies and other bioactive factors in human milk [16]. In developing countries, where access to clean water and safe breast-milk substitutes is often limited, these protective effects are especially valuable [16]. The combined benefits of maternal skin contact and breastfeeding likely contribute to the favorable infection profiles observed in low-hygiene environments.

Contemporary studies in low-hygiene, resource-constrained facilities across Africa and Asia consistently demonstrate that KMC maintains infection safety when basic hygiene protocols are followed. A 2021 randomized trial in Ghana showed improved survival among mild-to-moderately unstable low-birth-weight neonates receiving early KMC, with no increase in infection rates despite limited sanitation infrastructure [20]. A 2024 pilot study in India revealed favorable microbiome shifts in very preterm infants following KMC, with increased beneficial Firmicutes and reduced pathogenic Proteobacteria and Escherichia in suboptimal hygiene conditions [21]. A 2021 observational review in a Johannesburg KMC unit with high baseline sepsis risk and limited resources reported low mortality (3.1%) among very-low-birth-weight infants, attributed to KMC combined with simple hygiene practices [22]. A 2023 post-hoc analysis of the multicenter immediate KMC trial in low-resource hospitals (Ghana, India, Malawi, Nigeria, Tanzania) found lower rates of clinically suspected sepsis and sepsis-related mortality, addressing concerns in overcrowded, low-sanitation environments [23]. A 2021 systematic review in sub-Saharan Africa confirmed that integration of basic hygiene measures (e.g., handwashing and use of clean materials) effectively overcomes sanitation barriers to safe KMC implementation [24].

This review has several limitations. Heterogeneity in study design and outcome reporting prevented quantitative meta-analysis. Definitions of infection outcomes and hygiene practices were inconsistent, and long-term neurodevelopmental outcomes were rarely reported. Importantly, none of the included studies primarily evaluated hygiene conditions or specific hygiene interventions, limiting conclusions about the independent effect of sanitation on KMC outcomes. Although minor localized infections were occasionally reported, the available evidence does not indicate an increase in serious infections or infection-related mortality in low-resource settings.

Overall, the findings should be interpreted in light of the limited number of eligible studies and the heterogeneity in study designs, populations, and outcome definitions. While the available evidence is consistent in suggesting beneficial effects of KMC on neonatal mortality and selected clinical outcomes, the strength of evidence remains moderate rather than definitive. The findings should therefore be considered supportive, but not conclusive, and further well-designed studies with standardized reporting and clearer characterization of implementation contexts are needed to strengthen causal inference.

## Conclusions

KMC is a safe and effective intervention in low-hygiene and resource-limited settings. Evidence demonstrates reductions in serious infections alongside substantial neonatal mortality benefits. The implementation of basic hygiene measures further enhances the safety of KMC, supporting its widespread adoption in resource-constrained environments. Integration of KMC into existing maternal and newborn health programs can improve feasibility and long-term sustainability. Moreover, KMC's cost-effectiveness makes it a scalable solution for reducing preventable neonatal deaths in under-resourced health systems worldwide. Emerging evidence also underscores its role in promoting beneficial microbiome colonization, providing additional protection against infections in challenging sanitary conditions. Future implementation strategies should prioritize caregiver education and simple infection-prevention practices to maximize clinical benefits.
